# Sex, drugs and techno – a qualitative study on finding the balance between risk, safety and pleasure among men who have sex with men engaging in recreational and sexualised drug use

**DOI:** 10.1186/s12889-021-10906-6

**Published:** 2021-05-05

**Authors:** Nicklas Dennermalm, Julia Scarlett, Sarah Thomsen, Kristina Ingemarsdotter Persson, Helle Mølsted Alvesson

**Affiliations:** 1 Department of Global Public Health, Karolinska Institutet, 171 77 Stockholm, Sweden; 2 Department of Social Work, Stockholm University, Sveavägen 160, 113 46 Stockholm, Sweden

**Keywords:** Men who have sex with men (MSM), Chemsex, Recreational drug use, Harm reduction, HIV prevention

## Abstract

**Background:**

Recreational and sexual drug use among men who have sex with men may result in increased risk of poor health. The aim of this study was to better understand drug use and harm reduction techniques among Swedish men who have sex with men traveling to Berlin in order to improve the health of this population and inform public health strategies.

**Methods:**

A qualitative study based on semi-structured interviews with 15 Swedish men aged 23–44 with experience of drug use were recruited through network sampling. Interviews were conducted in Stockholm and Berlin and analysed using content analysis. The interview guide included questions on drug use, context, health and safety.

**Results:**

The participants engaged in drug use in both settings and in various contexts. Participants saw themselves as capable of finding a balance between pleasure, safety and risk with the aim to maximize positive effects while minimizing negative ones. The different risks of drug use were known, and participants relied on knowledge, harm reduction strategies and self-defined rules of intake to stay safe and healthy in a broad sense, both short term (i.e. during each session) and long term. Choice of drug and, frequency of intake, multi-use, risk of overdose, risk of HIV, purpose and context of use, how often, etc. were all part of the overall strategy. Knowledge of these methods was spread within the community and on-line rather than from counsellors or other health care providers. However, it did not always translate perfectly into practice and some had experienced overdoses and problematic use.

**Conclusions:**

The findings of this study point to the need for increased adoption of harm reduction techniques in this population focusing on mitigating harm and prevention of risk of problematic use or starting injection drugs. Existing traditional services require adaptations to become more accessible and acceptable to sub-groups of drug users, including low-threshold services providing non-judgemental, evidence-based information. This will require funding of alternative providers such as STI/HIV clinics, among others, and health care providers to increase adoption of prevention strategies, specifically pre-exposure prophylaxis for HIV.

**Supplementary Information:**

The online version contains supplementary material available at 10.1186/s12889-021-10906-6.

## Background

Recreational and sexual drug use in sex and/or clubbing venues among men who have sex with men (MSM) in Europe is a growing public health issue [1–3]. Recent data from the United Kingdom (UK) show that MSM are three times more likely to have used an illicit drug compared to heterosexual men in any setting [4], although one recent study reported a decrease in use in this population [5]. In a Swedish study with data from 2013, 7% of MSM had used recreational drug compared to 0.8% among the general population [6]. A more recent study from 2017 reported that 28% of MSM had experience in recreational drug use and 9% had drug use in combination with sex [7]. Recreational drug users across all populations represent an at-risk subgroup, but MSM drug users face specific challenges given the extent to which drug use is tied to the MSM social scene and pursuit of sexual partners [8]. Some research has explored characteristics of the users, i.e. time span, HIV status and sexual practice [9], marginalisation and minority stress [10] or exploring socio-sexual and other demographical characteristics [11]. Pleasure may act as a strong motive for drug use in this community, although it has been undervalued in favour of conceptualisations based on pathology and largely ignored within the framework of harm reduction [12]. ‘Pleasure’ and ‘rationality’ are concepts involved in drug use, especially when from a normative public health perspective pleasure becomes ‘hedonism’ and goes beyond ‘within reason’ [12]. On-line sexual drug use has also been shown to be a performative driver of pleasure [13].

The term ‘chemsex’ has been mainly used in a MSM setting and defined as “the intentional combining of sex with the use of particular non-prescription drugs in order to facilitate or enhance the sexual encounter” [14]. Such drugs include amphetamine, ecstasy/MDMA, GHB/GBL, crystal methamphetamine (crystal meth), ketamine, and cocaine [6, 14]. The term chemsex is closely tied with the gay social scene, with many clubs and saunas offering designated spaces to accommodate sexual activity on their premises [1, 8]. Having sex while under the influence of drugs in a party or club context is distinguished from intentionally combining sex and drugs as defined in chemsex [14]. Other terms to express the intentional combination of sex and drugs, such as “party and play”, have been used in Australian and American contexts [6].

Physical benefits of having sex on drugs include increased libido, sexual pleasure, and sexual longevity [14, 15]. Social and psychological benefits have also been shown to be motivators behind what some researchers call chemsex and others drug use, including facilitating sexual confidence (especially through improved body image and reduced fear of rejection), improving sexual intimacy, escaping one’s sexual identity and/or HIV status, and a sense of belonging in the gay community [16]. Having a social interaction with other gay men has been associated with stronger endorsement and a lower perception of risk [17]. The introduction of technology in the form of geolocation dating apps are contributing to the increased visibility and accessibility of chemsex [1]. Studies have shown associations between MSM who use drugs and poor health outcomes, including overdose, death, needle-sharing transmitted infections and mental health problems [1, 18–20]. Several studies also indicate an association between drug use and high-risk sexual behaviour from an HIV perspective [6, 8, 21–26], though there is no consensus on the strength of the association [6, 27]. MSM using drugs or travelling abroad have been shown to take sexual risks, such as anal sex without a condom with a non-steady partner [6, 28]. One study highlighted the association between chemsex and STIs in HIV negative men, but not in HIV positive men [29]. A higher prevalence of recreational drug use in MSM was found to be associated with having multiple sexual partners and anal sex without a condom with a non-steady partner [6, 24]. There have also been indications that risk-taking behaviour and resulting poor health outcomes are increasing among drug using MSM [1, 2]. In particular, recent British research has highlighted increasing overdoses and deaths related to GHB/GBL, a problem which is a likely result of the increased use of the drug in the chemsex scene in London [15]. Some London clinics have also reported an increase of intravenous crystal meth in combination with sex among MSM [15]. Vulnerability to sexual assault has been reported when being on drugs in some studies [1, 20] and correlations between chemsex and sex work have also been found [24]. Thus, there has been a call for increased harm reduction efforts targeting this community [20, 23, 30–32]. These men are not hard-to reach populations since they can be reached in traditional STI testing settings [1, 25, 26, 33, 34] or non-governmental organisations catering for the MSM community [35].

Travelling to larger or cities with a reputation of being more sexual liberal has become a common feature in the lifestyle of Swedish and other European MSM, with nearly a third of Swedish MSM reporting engaging in sex while abroad within a six-month recall period [28]. In our prior study exploring sexual practice among Swedish MSM travelling to Berlin, we found that 15 out of the 18 participants had experience of drug use in both cities [34] and the data are currently being analysed. This sub-study was situated in Berlin to explore recreational and sexual drug use in Swedish MSM, although that was not the original purpose of the data collection.

Sweden is interesting to investigate from a health perspective due to its institutionalized zero tolerance approach to illegal drugs and given that drug users are criminalized. Harm reduction policy and methods are key in order to ensure health and human rights for drug users. Historically, from a policy perspective Sweden has chosen to adapt and promote a policy model that makes no distinction between cannabis and other illicit drugs, i.e. cocaine. As a result, harm reduction is not promoted widely since it is perceived as a contradiction towards achieving the goal of a drug-free society [35, 36]. However, a Swedish policy debate during the 2000s led to the establishment of national needle exchange programs in order to combat HIV and hepatitis transmission [37]. In many other countries harm reduction is adopted because of its evidence of contributing to better health outcomes as well as a human rights-based practice [38–40]. Germany has a less punitive approach than Sweden where use and possession for personal use is decriminalised [41].

The aim of this study was to improve understanding of drug use, sex on drugs and harm reduction techniques among Swedish MSM who travel to Berlin in order to improve health among MSM using drugs.

This study describes a group of MSM traveling between two very different contexts of sex and drug use in Berlin and Sweden, with a range of drug-using experiences represented - recreational drug use, sexual drug use more broadly, and chemsex. 

## Method

### Study design

This study is part of a larger project exploring HIV/STI risk behavior and prevention among highly-sexually active Swedish MSM who have sex in Sweden and Berlin [34]. The study presented here explores a subset of the experiences of Swedish MSM in this population, focusing on recreational drug use and/or sexual drug use. Data collection was conducted January 2016 to June 2017 using semi-structured interviews. We did the interviewing in Stockholm and Berlin, both face-to-face and via video teleconference depending on the location of the interviewer and the participants.

### Participant selection

Network sampling was used for recruiting 15 study participants [42]. Following this methodology, recruitment was generated from five seeds within the interviewer’s broad network while aiming to avoid seeds from the same circle of people. These five provided referrals for participants. However, four of the five seeds were also interviewed as participants. The fifth seed used to work with sexual health and were excluded for bias reasons. The eligibility criteria were: i) Swedish citizen, ii) cis-gender men who have sex with men, iii) aged 18–46, and iv) currently or formerly a resident of Berlin or travels to Berlin at least twice per year, and v) recreational drug use or sexual drug use during the past 3 years. Each participant was thereafter asked to contribute a maximum of two referrals to minimize the risk of community bias. The full participant profile is presented in Table 1. The transcripts were analysed in parallel with the data collection. After the 11th interview, we started to hear increasingly similar stories on a number of topics like how to control intake or the importance of balance. New codes were still developed but no new sub-categories were developed. We decided to continue the interviewing as long as the waves continued.

**Table 1:** Profile of the participants in the study

Demographic Variable	***N*** = 15
*Age*	
20–29	5
30–39	7
40+	3
*Education*	
High school or less	3
Vocational training	2
Attended university	10
*Employed*	
Yes	13
No	2
*Relationship status*	
Non-monogamous relationship	6
Monogamous relationship	0
Single	9
*Reported HIV Status*	
Positive	1
Negative	14
*HIV and/or STI testing last 12 months*	
Yes	15
No	0
*Using Pre-Exposure Prophylaxis (PrEP)*	
Yes	1
No	14

### Participant characteristics

The 15 participants ranged in age from 23 to 44. Most were currently employed and had a university education. The most commonly used drugs were ecstasy/MDMA, amphetamine, GHB/GBL, cocaine, ketamine, cannabis and to a lesser extent, methamphetamine. They had high numbers of sexual partners and the majority of the men had experienced sex in clubs/saunas, group sex and open relationships. Berlin was the prime travel destination for the men who did not live in Berlin.

### Data collection

Data were collected using semi-structured interviews. The same interview guide was used as the one for the parent study, which was inspired by topics from the 2013 cross-sectional survey of MSM living in Sweden and included a section on drug use [43]. An English translation of the interview guide is available as a supplementary file. The unit of analysis in this sub-study was content related to the topics of drug use. Data on other topics including travelling, access to HIV/STI preventative services, sexual risk-taking, sexual practices, and HIV/STI screening behaviours and experiences were collected in the interviews but analyzed and presented in a previously published article [34] and in a manuscript yet to be published. The guide was piloted with three participants. Since no crucial revisions were made to the guide, the pilot interviews were included in the sample. All interviews were conducted in Swedish by the first author, either face to face (*n* = 10) or through video teleconference (*n* = 5) and recorded after receiving informed consent to allow this. Interview recordings were transcribed verbatim by the interviewer and a contracted professional transcriber. Interview duration ranged from 45 to 170 min.

Given the sensitivity of the study topic, efforts were made to build trust with the participants. While social desirability bias is difficult to eliminate, we assess that it was minimized by using an interviewer with understanding of the culture. The interviewer, an openly gay Swedish man, had extensive experience working with MSM, drugs and sex work and was able to pose the questions using a respectful tone and avoiding moral judgement. The issue of social desirability bias was addressed verbally before each interview in order to reduce bias. The interviewer travelled to Berlin from February to May 2016 to conduct data collection and returned July to August 2017 to continue writing. He kept a research diary with immediate reflections after each interview and shared it with the author team on a weekly basis, including reflections on method, progress and analysis. Participants appeared open and willing to share many examples from their own lives and talked about successful strategies, failures and illegal activities.

### Analysis

Qualitative content analysis was used to analyze the 15 transcripts [44], supported by NVivo data analysis software. The interviewer (ND) developed a list of 305 codes while collecting the data in close collaboration with a second researcher (KIP). Any discrepancies were addressed and negotiated during weekly meetings until consensus was reached. Then the codes were organized into sub-categories, categories and themes. ND and KIP later shared the codes with an additional researcher (JS) who read the transcripts several times and was familiar with the content. Two senior researchers (ST early in the process and HMA later in the process) supervised these discussions and the iterative code development process. To facilitate continuous discussion on the large number of codes among team members, the final part of the analysis was conducted in Microsoft Excel. No changes were made in the clustering during this time.

### Ethical considerations

This study was approved on February 11, 2016, (2016/32–31) by the Regional Ethical Review Board in Stockholm. Participants were provided information regarding the study, data storage and the conditions of their participation prior to consenting to be interviewed. In recognition of the sensitivity of the interview topics, participants were provided contact information of a counsellor in Sweden, a telephone hotline and a web-based peer support service in case they wanted to discuss anything further. As compensation for their time, participants were given two movie tickets. No additional compensation was provided to participants who made referrals. 

## Results

The academic definition of chemsex highlights how specific drugs are intentionally used in order to heighten or enable certain sexual practices. The term was used by some participants but not all.

When it has been chemsex, it has always been some kind of … I don’t know … a group [sex] thing. Something like that. Yeah, sure, there has been sex while being high, but for me it’s more that you arrive home and you are already high and you may have some [drugs] left. And I’m like ‘God, I have a small line left, should we?’” (#10)

This was the case in a variety of settings, at a techno club, spontaneous hook-ups or afterparties.

The participants’ experiences were largely described in relation to the concept of balance. While maintaining balance between pleasure and safety was commonly noted, so was the ever-present possibility of tipping the scale too far and experiencing negative effects. Participants’ experiences and behaviours were influenced by (i) positive and negative effects of drug use; (ii) conscious drug choice; (iii) contextual aspects of drug use (iv) risk concerns; and (v) harm reduction.

### Positive and negative aspects of drug use

The men expressed a broad range of both positive and negative aspects associated with drug use. All men expressed elevated club and sexual experiences due to drug use, including heightened physical pleasure and the possibility of longer sessions, as well as psychological benefits such as increased confidence and wellbeing.

I don’t take drugs to escape something. I take drugs in order to elevate an experience. Like when you are out clubbing. It feels like you are levitating […] you get intoxicated […] then the music, the lights, the people. (#03)

For others, drugs were used to escape negative thoughts or just to free their minds from inhibitions, resulting in a better social experience, e.g. more sexual partners and new sexual practices like punch fisting (#10) and sex with women (#05). Participants often described the experience of using drugs as unparalleled to the same experiences without drugs. Words used by the participants included a sense of “flying” on crystal meth (#08), “euphoric” and “affectionate” on MDMA (#04) and feeling like a “disco diva” on cocaine (#13).

However, most participants who used drugs experienced or were concerned with the negative effects during and/or after a period of drug use. These ranged in severity from dizziness, stomach problems and impotency to risk of HIV transmission, rape, problematic use, overdose and death. In particular the risk of overdose was a topic that worried the participants, stemming from both their own and their friends’ or partners’ experiences. (see the section ‘Harm reduction’.) Participants also commonly mentioned the impact of their drug use on their mental health, with some mentioning periods of anxiety and panic after partying. While some felt that drugs impacted their social lives positively, others mentioned drugs had caused them to feel lonely and to even lose friends or family.

“It’s like a little ticket to heaven in five minutes. You can’t get so high so fast and have so much fun in your everyday life ( …) You can hardly get that in any other way than by using drugs. But it will cost you. ( …) The beauty of a lot of other things fade. Why should you stay at home on a Saturday having dinner with your family when you can be dancing in heaven and just feel extremely well? Having that much fun can be dangerous since everything else becomes so much more boring.” (#11)

Compensatory positive aspects were also mentioned, such as using Viagra to offset drug induced temporary erectile dysfunction or amphetamines to offset alcohol induced tiredness and/or GHB/GBL overdoses. The different aspects and purposes of the drugs were the reason many informants reported multidrug use.

### Intentional and personalized choice of drugs

Drug choice was a key aspect for the men to maintain balance between risk, safety and pleasure. While the price of some drugs varied depending on the location (Berlin or Sweden), there was no indication that price was a crucial factor in the men’s choice of drugs. Instead, drug preferences were commonly based on which drugs would provide maximal benefit and minimal negative effects. Previous experience with certain drugs was therefore a driving factor behind their drug choice. The men were aware of the wanted and unwanted effects of drugs they had experienced and made intentional drug choices to maximize their personal preferences based on this information. Men were also intentional about the frequency of using certain drugs, a choice that also sought to maintain an optimal balance. In this regard, participants mentioned avoiding drugs that had unpleasant side effects, preferring other drugs instead or saving such drugs for special occasions only.

“You cannot cope with being high on some of these drugs too often, because you become crazy afterwards. It takes too much energy like, physical and mental. And it’s often not fun. So, some drugs are only fun very, very seldom.” (#06)

The men also recognized that their mood at the time of the drug use also affected their preference, and by monitoring how they felt they selected drugs that would produce the desired effect or decided not to do drugs in that particular moment.

“[It’s important] that you feel overall fine before taking anything. That your energy flow is good. [ …] It’s like when you take something, everything amplifies. It’s the same with alcohol, you get extra happy on it. But if you are [not in a good place], you’re ok to go out, you’re hanging around with people, but then you don’t reach the magic moments. And then you just have to accept it, but that is when many people make a mistake. They don’t get the kick they search for, so they take a little bit more and [overdose].” (#03)

Drug choices were also based on the type of activity they would be engaging in. For example, participants consistently stated that GHB/GBL and methamphetamine were drugs for sexual purposes, and particularly highlighted the popularity of GHB/GBL among men who included fisting in their sexual repertoire due to its muscle relaxing effects. Despite the men having high autonomy in their drug choices, local availability of certain drugs and the contacts the participants had for accessing drugs also influenced the choice of drugs.

“I don’t think I have taken G in Berlin. I have taken ecstasy at clubs though, because it was really easy to find there. All you had to do was ask someone …” (#07)

### Contextual aspects of drug use

Overall the men reported similar frequencies and quantities of drug use while in Berlin and Sweden. However, there was a consensus among the men that Berlin offered a more liberal and open drug/club compared to Sweden.

“It’s very open, the drug scene. There are a lot of drugs in Berlin. But there are a lot of drugs in major cities, and especially in the gay world. ( …) But there is an openness among the people in Berlin, there is less hush hush about it.” (#13)

Some men felt that their drug use habits varied as a result of the different clubbing experiences. Clubs in Berlin were viewed as superior to those in Sweden, both in experience and opening hours. During the interviewer’s stay in Berlin it was noted that Berlin clubs could stay open for entire weekends, whereas it is more common for clubs in Sweden to have closing times. Some felt that as a result there was no need to take drugs that would prolong party stamina in Sweden.

“Then again, I haven’t been out clubbing in Sweden either, I don’t have the same need to go out dancing until 10 in the morning because you almost can’t do that here, even if you wanted to. Then you don’t need any drugs.” (#02)

However, for some the perceived lower-quality club scene in Stockholm meant that drugs became the main activity of the night instead of clubbing. One participant expressed that when taking drugs at one’s home there was no clear start and end, whereas at clubs, either with closing hours or not, there was a certain point in time when one felt a desire to stop and go home.

While many of the participants had sex under the influence of drugs, not all had experience with chemsex, as in taking drugs with the intention of having sex. Rather, some participants used drugs in club and social settings to improve the experience, and sex was a positive and sometimes expected and appreciated by-product. In this way, drug use, clubbing, and sex were all closely linked.

“We have already taken something, we are a little bit horny, here we go! You know, a party could happen, an orgy could happen. You know, whatever. It can be one on one, it can be whatever. It becomes whatever you feel like doing.” (#10)

### Risk perception when using drugs

Several participants were aware of the risks involved in their drug and/or sex habits, and were concerned with balancing risk with personal safety, albeit not always maintaining such balance. Risk concerns included extent, frequency and control of their drug use, difficulty moderating drug use (i.e. going from “one extreme to the other”) the use of drugs as an escape from reality, losing track of time while taking drugs (especially GHB/GBL and crystal meth), dosing and overdosing, risk of acquiring HIV and other STIs or relying fully on drugs in order to enjoy sex.

One of the largest concerns among the participants regarding sex in general and on drugs specifically was the risk of acquiring HIV or other STIs, mainly through sex but there was also a concern about needle transmission when injecting drugs.

“I get HIV related anxiety, or STI anxiety. [ …] My doctor said: ‘Great that you get tested because you can get the Big Five in one single screw [in Berlin]’. And with The Big Five he meant gonorrhoea, chlamydia, HIV, syphilis and hepatitis.” (#05)

Others expressed less fear of becoming HIV positive now. “I’m not as afraid of |becoming HIV positive] as I was ten years ago. I know many who are living with it and do not have any problems with it. [ …] And they cannot pass it on” (#09) Despite attempts to adhere to safer sex, a tension between safe sex and the experience was often noted. A few participants felt that drugs made it difficult to adhere to safe sex practices.

“And then that damn sense of happiness occurs [when doing drugs]. It’s like six, seven, eight, nine, ten, eleven, lunch time when the party is kind of fading. But you are still happy. You have been dancing with someone for maybe two hours. And then you go home and you have sex. And then it is where I think it becomes risky, just because I am so happy. It’s like underwear off! Flower power! Off with your clothes and you dance at home, and you’re naked and oh-la-la [ …] but that is where there is also an increased risk taking. But there have also been situations where we have started having sex and suddenly realized: ‘Wait a minute. We are not using a condom. We should [use a condom]’.” (#04)

PrEP was not widely available neither in Germany nor Sweden at the time of the data collection, which could explain why only one participant was on PrEP. Yet there was generally a positive attitude towards PrEP among the participants. The main reason for not using a condom was pleasure and allowing the sexual experience to take priority over risk concerns in the moment of action. All participants in this study were tested for HIV and/or STIs one to four times per year, and knowing one’s HIV status was perceived as important. The vast majority of the participants visited the same MSM clinic in Stockholm and were satisfied with the service due to the availability of an easy, full screening service and a non-judgmental attitude among the staff. Some of the participants who lived in Berlin still preferred to visit the clinic while visiting Sweden.

Overdose was a major risk that the men tried to avoid through a carefully controlled intake of drugs. This was both a personal and a community concern, with one participant warning specifically about GHB/GBL: “… the tricky part for some guys, for many people is dosing, there is a fine line between using and overdosing.” (#10). Another informant described how he on several occasions had helped men he did not know who had overdosed to make sure that they did not suffocate on their own vomit or pass out. This is linked to the concern about other men’s lack of drug use knowledge, especially regarding GHB/GBL. The participants felt that people often confuse the two, which could increase overdose risk because of differences in the dosage of each drug.

The risk of becoming ‘addicted’ to drugs was discussed by several participants, but mainly regarding the risk of relying on drugs in order to enjoy sex rather than a conventional perception of addiction. While most participants had both sex without the influence of drugs and sex on drugs, some had lost this balance. One participant elaborated on his struggle to reconnect to a sexuality without the influence of drugs after experiencing sex on drugs.

“Sex on drugs was so amazingly free and almost pornographic. It gets so intense. A drug, what it does is open a tap within your mind. And then having sex simultaneously becomes very, very intense and pleasurable. [ …] It’s taken a long time to think that sex is as interesting without drugs.” (#11)

Others were more concerned about this risk at the community level.

“Often there are shy guys who tried chemsex, and then they cannot stop with it because it is the only way they think they can have sex. They never developed some self-esteem to just flirt or talk with guys and stuff normally.” (#10)

### Harm reduction

Aligned with the theme of maintaining balance between risk, safety and pleasure, participants employed several methods to facilitate this balance. They usually learned from more experienced drug users, various online resources or through personal experience. These methods included ways to minimize harm short term (i.e. one session or weekend) or long-term. In Table 2 these techniques have been clustered into four categories: controlled intake, knowledge about drug use, pre-decided behaviour rules in order to maintain health long-term, and strategies for handling overdoses.

**Table 2:** Reported harm reduction techniques during drug use and/or sex on drugs

Harm reduction techniques	
Controlled intake (short-term management)	• Identify correct individual dosing. • Dosing schedules • Tracking dosing using mobile phone • Synchronizing dosing with friends • Avoiding mixing drugs and alcohol or other perceived harmful combinations of drugs
Knowledge about drug use	• Learning about drug effects on the body • Learning how to take drugs from experienced friends • Learning the difference between GHB and GBL • Being aware that the body responds differently depending on the drug used, quality of the batch, etc.
Pre-decided behaviour rules in order to maintain health long-term	• Only take drugs with friends • Alternate drug us weekends with drug-abstaining weekend • Drug use frequency (i.e. only once per month) • Taking drugs only recreationally • Having chemsex only with people they are comfortable with • Avoiding to use drug with every sexual partner • Not injecting drugs • Not to share injecting tools • Avoiding drugs for sex
Strategies to handle overdoses	• Looking out for each other, club families • Use of amphetamine to counteract overdose from GHB/GBL or alcohol • Drinking water if overdosing

For many, correct dosing and control over the strength of the substance was key when addressing the risk of short-term danger. Correct dosing was the main way to control their drug intake and maintain their perceived safety balance. In the example of GHB/GBL, the participants measured their intake carefully and used mobile phones to keep track of intake intervals in order to stay safe while at the same time heighten the experience. Some of the participants had experienced not being in control of the dosage either recently or in the past, and of not following their own methods. Some participants also expressed the importance of being knowledgeable about the drugs they used, which included effects, strength, different batches or manufacturers, potentially dangerous combinations and variations among close-related drugs like GHB vs GBL.

The common use of self-defined behavioural rules as harm reduction techniques demonstrated that the men’s perception of balance between risk, safety and pleasure was personalized. Personal behavioural rules included balancing their drug use with their normal lives, either by mixing drug weekends with drug-abstaining weekends, only using drugs once per month, or maintaining routines. These rules or street-smart know how were being passed around within the community. One participant described his initiation to drugs like this:

“He’s like my little brother. He taught me how to do drugs. What to think about, what to be careful about and how to mix. […] I didn’t know. Even if you’ve read something on a drug website somewhere on how different drugs work, even then, you need someone to explain it to you.” (#03)

While controlled intake addressed short term aspects of drug use, behaviour rules were addressed more long-term health issues. Several participants elaborated on the need for structure in everyday life in order to be able to have a long-term healthy life while still using drugs. It was a common belief that those who did not have this structure were more easily caught in problematic use and moved back to Sweden. One participant stated:

“It is important to have something, so one has a balance between this crazy party world [ …] it doesn’t need to be a nine-to-five job. Outside of it you just go to the gym, you do stuff … you engage yourself in your day.” (#03)

Even though the participants did not deliberately created structure in order to avoid problematic use, it was clear that they saw structure as a behavioural rule in order to keep drug use under control. Other behavioural rules involved complete avoidance of aspects the participants felt to be very dangerous or undesirable. Injecting, or slamming as it is called within the chemsex scene, was a threshold many did not want to cross since it was viewed as being more severe or dangerous. Some men also had behavioural rules regarding sex on drugs, such as only having sex partners they are comfortable with drugs or not having sex on drugs routinely.

Some participants were aware of community remedies for dealing with overdoses, which illustrates their perception of self-control in balancing pleasure with risk. For example, amphetamine was mentioned as a key remedy to help those who are overdosing from GHB/GBL.

“You just have to go around and ask for speed and try to get the person to take it, because it usually helps. Amphetamine usually woke people up. It’s a little like a home remedy that people use at techno clubs … It takes five minutes and the guy is back.” (#13)

Participant 10 had personally experienced taking too much GHB/GBL but was able to avoid passing out by snorting amphetamine. He also had witnessed an unconscious man regain consciousness after being administered a small dose of amphetamine rectally by another more experienced club-goer. The importance of community and friends was stressed as a key factor for dealing with an overdose safely.

“And it’s like you have friends and they take care of you. It is a lot of that in the club culture and especially at [popular techno club in Berlin] on Sundays. You take care of each other … I have taken care of plenty of people that have like passed out.” (#04)

None of the participants mentioned calling an ambulance or other medical help in the event of an overdose. All the participants relied heavily on personal experience or community knowledge rather than on information or guidance from the medical profession, non-governmental organizations or similar actors.

## Discussion

This study found that Swedish MSM with experience of recreational and sexual drug use attempt to create a balance between risk and pleasure by integrating a variety of safety measures into their practice. Participants saw themselves as capable of controlling risk through intentional and personalized choice of drugs, leading them to make deliberate choices they felt would minimize negative side effects and maximize positive effects. Even in varying contexts (e.g. Sweden versus Berlin, club versus home party) the men expressed concerns about risk, and this risk perception resulted in different types of harm reduction techniques. Often their notion of balance was based on maintaining self-defined behavioral rules. The development and application of behavioral rules in this way have been noted in other studies in MSM who use drugs and/or have sex on drugs [35, 45]. They are part of a Berlin community which reshapes the outer boundaries of ‘pleasure’ and ‘responsibility’ in the search for what O’Malley and Valverde (2004) call ‘hedonism’ but without a total loss of control. The Berlin lifestyle can be considered as a kind of ‘hedonism’ but still maintaining what could be labelled in Sweden as a behaviour ‘within reason’ [12].

The dominant reference to community knowledge may signal that wide-spread and evidence-based harm reduction techniques are not communicated effectively or discussed in professional settings. A previous study correlated with our findings in that knowledge is being passed down by the “chemsex circle of experts and novices” [20]. This may explain the lack of professional harm reduction techniques noted in the men’s experiences, such as calling an ambulance for someone who is overdosing, discussing sex on drugs with their physician or use of PrEP. Other professional harm reduction techniques include professional support, psychotherapeutic services, and informational websites which have been shown to be underutilized, mostly because men felt the services would not be able to appropriately discuss MSM health and drug use [15]. None of the men mentioned seeking help to quit drug use, although some expressed concerns with possible problematic drug use.

The level of perceived self-control exhibited in the MSM who used drugs recreationally or sexually in this study is distinct from other populations of drug users. For example, whereas low-income drug users in the United States use crack cocaine because it is less expensive [46], drug price was not a key determinant for our study participants. This may reflect both the socio-economic characteristics of the participants, who were employed and highly educated, and the recreational use of drugs rather than daily use. Additionally, no major difference in the men’s behaviors in Berlin versus Sweden was reported, whereas current literature demonstrates increased risk taking in MSM during travel [28, 47]. However, one study found that MSM may take fewer sexual risks when travelling internationally as compared to domestically [48]. It may be that the men in our study population feel equally comfortable in Berlin as in Sweden, given that they had lived in or visited both places for extended periods of time. This may indicate that men with high self-awareness and risk-perception, which may also come from experience, have been able to choose to condition themselves to maintain their personal balance between risk, safety and pleasure regardless of context.

There were men in our study who had experienced unplanned anal sex without a condom and felt it was a result of their drug use, demonstrating that their perceived capability of maintaining balance does not translate perfectly into action. Even though there was little evidence supporting the correlation between drug use and anal sex without a condom in this study, the potential negative influence of drugs on condom use has been shown in other studies [6, 8, 21, 22] demonstrating the increased risk of acquiring HIV by having sex under the influence of drugs. Low PrEP use in this study could be attributed to the lack of availability through the Swedish and German health care systems at the time of data collection. The sexual risk perception among these participants will be explored in a future manuscript.

Our analysis revealed that recreational drug use was closely linked to clubbing, where sex was an often expected and appreciated by-product, though not always the intention. In this way the mens’ experiences did not always fit the definition of chemsex as used in previous literature where drugs are used intentionally for heightening the sexual experience [1, 14, 45, 49]. This is an important finding that should be considered when targeting men for harm reduction efforts, as it may be that MSM who are clubbing would not consider themselves as part of the chemsex scene but are exposed to similar risks if having sex as a by-product of their clubbing.

Community knowledge on drug use and safety can benefit the users and should be taken into consideration when designing interventions. With that said, one alarming finding of this study is the lack of professional harm reduction guidance among the men we interviewed. Despite contact with the health care system for STI and HIV testing, they learned from various non-specified websites, people within the community and from their own experiences. This is not necessary a negative thing. However, this may hinder MSM who will use drugs in the future, MSM who are about to drugs for the first time or men without a connection to the community from making informed decisions about their health and lives.

### Methodological considerations

While the study population is limited to Swedish MSM who travel to Berlin, the study results could be transferrable to other European MSM as well as to those who seek partners in Berlin since these men are part of a larger European community of MSM travelling to Berlin for reasons of freedom, sexual adventure and drug use. The idea of Berlin as the pan-European capital of decadence was explored in our previous study. However, socially and culturally, the experiences of drug use in Berlin and Sweden would likely differ from other large MSM destinations such as England, Spain, and Thailand since the variation in cultural settings would create a different framework for drug use.

The travelling pattern among these men differs from that reported in other research, as they returned to the same city repeatedly or even decided to move there. This makes comparisons with other studies focusing on traditional vacations more difficult [13, 24]. It is important to note that our participants were not daily drug users and typically only used drugs in social, sexual or clubbing contexts. Further research should explore if and how experiences of MSM engaging in chemsex to see how and if their experiences differ from those using recreational drug use.

### Limitations

When we conducted the parent study on travelling and sexual behaviour, recreational and sexual drug use were included in the interview guide but given a limited space during the interview. This itself creates limitations regarding depth, regarding for instance exploration of the different policy contexts and their impact on health and health services, as well as specific on-line drug use. In addition, we used existing interview material where only some of the participants fit the narrower definition of experiencing chemsex. This led us to explore sex and drug use more broadly, rather than the narrower term and behaviour of “chemsex.” This reflects the context for many of the participants who are travelling between different penal systems and also between different sexual and drug practices. We therefore analyzed a larger group of MSM using recreational drugs. While our study provides insights into sex and drug use that are likely similar and applicable to men engaging in chemsex, a larger sample would allow for additional comparisons of knowledge and harm reduction techniques between those who have had chemsex according to the narrower definition compared with those who did not fit the definition but still had experience of sexual drug use. Other limitations include age span, lack of data based on socio-economic factors and limited amount of men with experience of injecting drug use.

## Conclusion

The men in this study sought to maintain a balance between risk, safety and pleasure. They perceived having a sense of autonomy and control over their drug use and sexual habits, though they did not always maintain balance or safety. Harm reduction techniques were used to combat risk concerns regarding both drug use and sex on drugs, most of which were learnt from their own or friends’ experiences, not via professional interventions.

This study points to the need for increased adoption of harm reduction techniques in this subgroup of MSM. These efforts should focus on mitigating harm as well as prevention of further risks, i.e. of becoming addicted or starting to use injection drugs. Public health efforts should consider ways to bridge the gap between the community and different health care actors in preventive and acute care. This includes both the non-professional methods used in the community as well as professional methods, particularly PrEP [50]. We urge advances in policy and public health efforts to make PrEP easily accessible for MSM and especially for MSM using drugs. Furthermore, there is a need for greater counselling of evidence-based and professional prevention tools on behalf of service providers in HIV/STI clinics and other relevant settings in order to increase adoption of these strategies by MSM. Interventions should be made in collaboration with MSM using drugs and interventionists to design harm reduction strategies that are accessible and acceptable. Qualitative research is needed to fully understand all aspects of recreational and sexualised drug use, including users’ attitudes and health concerns within different legal systems, in order to inform tailored health programs for this target group. More research is also needed to understand the user’s attitudes and health concerns in different legal systems.

## Supplementary Information

**Additional file 1.** Interview guide.

## Data Availability

No primary data is available due to the sensitive nature of the content and aligned with the application to the Ethical Review Board in Stockholm in order to ensure the anonymity of the participants. A translation of the interview guide is available as a supplementary file.

## Abbreviations

Bottom: Refers to men who have sex with men and who prefer to be penetrated when having anal sex.
Chemsex: Sexualized drug use within men who have sex with men. This term is usually used when writing about drugs being used specifically for sex in order to enhance the experience of enabling certain practices when muscle relaxation is needed.
G: Short for GBL or GHB
GBL: γ-butyrolactone
GHB: γ-hydroxybutyrate
Fisting: Sexual practice when a fist is being inserted into anus or vagina. Punch fisting refers to a practice where the fist was inserted with force and/or speed
HIV: Human immunodeficiency virus
MDMA: 3,4-methylenedioxymethamphetamine
MSM: Men who have sex with men
STI: Sexually Transmitted Infections
Party n’ Play: Slang for chemsex sessions
PrEP: Pre-exposure prophylaxis for HIV
Sex on drugs: When having sex while being on drugs. However, the sex was not the purpose of the intake. Compare with ‘chemsex’
