# Supplementary material for: Sex, drugs and techno – a qualitative study on finding the balance between risk, safety and pleasure among men who have sex with men engaging in recreational and sexualised drug use
Source: BMC Public Health. 2021 May 5;21:863. doi: 10.1186/s12889-021-10906-6 (PMC8097859; doi:10.1186/s12889-021-10906-6)
Supplement: Supplementary file 1 — Additional file 1. Interview guide. [file 12889_2021_10906_MOESM1_ESM.docx]

**SUPPLEMENTARY FILE: INTERVIEW GUIDE**

**INTRODUCTIONARY QUESTIONS**

Introductory questions to the participant on demographic information.

**BERLIN IN COMPARISON TO SWEDEN**

What do you like about Berlin as a destination to visit?

What were your expectations initially and have they changed over time?

Describe the dating scene in Berlin. How do you meet potential partners in Berlin vs Stockholm?

How to you perceive dating and the sex culture in Berlin vs Sweden? What kind of sex do you take part in?

**HIV AND SAFER SEX**

Can you explore your thoughts about HIV and STIs, both in Berlin and Sweden?

How do you perceive and practice safer sex and condom use today compared to earlier in life? In Berlin vs Sweden?

How do you relate to risk reduction strategies beyond condom use?

Are you familiar with PrEP?

How do you perceive the effectiveness of PrEP?

Would you consider using it yourself??

Have you ever got a HIV or STI test?

Have you tested positive for an STI and if so, how did you experience that?

What were the reasons for your last test?

When and where did you take your last test?

**ALCOHOL AND DRUGS**

How do you look upon alcohol use in Berlin vs Sweden?

Do you drink alcohol?

How do you look upon drug use in Berlin vs Sweden?

Do you use drugs? Please describe purpose, context and consequence.

Any thoughts about poppers in a sexual context?

Do you use poppers? Please describe purpose, context and consequence.

**END QUESTION**

Would you like to share anything else that may have relevance for this project?
